# A phylogenomic study of Steganinae fruit flies (Diptera: Drosophilidae): strong gene tree heterogeneity and evidence for monophyly

**DOI:** 10.1186/s12862-020-01703-7

**Published:** 2020-11-02

**Authors:** Guilherme Rezende Dias, Eduardo Guimarães Dupim, Thyago Vanderlinde, Beatriz Mello, Antonio Bernardo Carvalho

**Affiliations:** Departamento de Genética, Universidade Federal Do Rio de Janeiro, Caixa Postal 68011, Rio de Janeiro, 21941-971 Brazil

**Keywords:** BUSCO, Incomplete lineage sorting, Introgression, Species tree

## Abstract

**Background:**

The Drosophilidae family is traditionally divided into two subfamilies: Drosophilinae and Steganinae. This division is based on morphological characters, and the two subfamilies have been treated as monophyletic in most of the literature, but some molecular phylogenies have suggested Steganinae to be paraphyletic. To test the paraphyletic-Steganinae hypothesis, here, we used genomic sequences of eight Drosophilidae (three Steganinae and five Drosophilinae) and two Ephydridae (outgroup) species and inferred the phylogeny for the group based on a dataset of 1,028 orthologous genes present in all species (> 1,000,000 bp). This dataset includes three genera that broke the monophyly of the subfamilies in previous works. To investigate possible biases introduced by small sample sizes and automatic gene annotation, we used the same methods to infer species trees from a set of 10 manually annotated genes that are commonly used in phylogenetics.

**Results:**

Most of the 1,028 gene trees depicted Steganinae as paraphyletic with distinct topologies, but the most common topology depicted it as monophyletic (43.7% of the gene trees). Despite the high levels of gene tree heterogeneity observed, species tree inference in ASTRAL, in PhyloNet, and with the concatenation approach strongly supported the monophyly of both subfamilies for the 1,028-gene dataset. However, when using the concatenation approach to infer a species tree from the smaller set of 10 genes, we recovered Steganinae as a paraphyletic group. The pattern of gene tree heterogeneity was asymmetrical and thus could not be explained solely by incomplete lineage sorting (ILS).

**Conclusions:**

Steganinae was clearly a monophyletic group in the dataset that we analyzed. In addition to ILS, gene tree discordance was possibly the result of introgression, suggesting complex branching processes during the early evolution of Drosophilidae with short speciation intervals and gene flow. Our study highlights the importance of genomic data in elucidating contentious phylogenetic relationships and suggests that phylogenetic inference for drosophilids based on small molecular datasets should be performed cautiously. Finally, we suggest an approach for the correction and cleaning of BUSCO-derived genomic datasets that will be useful to other researchers planning to use this tool for phylogenomic studies.

## Background

The Drosophilidae family is traditionally divided into two subfamilies, Drosophilinae (~ 3,500 species) and Steganinae (~ 700 species) [1]. While many Drosophilinae species have been widely studied (e.g., *Drosophila melanogaster*, *D. pseudoobscura*, *D. mojavensis* and *D. virilis*), the Steganinae subfamily remains poorly understood [2]. Scarcity of data about their ecology, development, taxonomy and phylogenetic relationships can be explained by some characteristics of Steganinae: most species have peculiar life habits (e.g., predatory species and parasites), are not attracted to the usual fermented fruit baits, and are hard or impossible to breed in the laboratory [3, 4].

The traditional division of Drosophilidae into two monophyletic subfamilies was suggested by morphology-based studies [5–7] and is widely accepted (e.g., [4, 8–10]). However, no single exclusive diagnostic morphological character distinguishes Drosophilinae from Steganinae [4, 7, 11]. Furthermore, in contrast to morphology-based studies, molecular phylogenetic studies have recovered Steganinae both as a paraphyletic [12–15] and as a monophyletic [16] clade. Related to this, no study to date has addressed the ancient divergences within the family using a phylogenomic approach, which is arguably a powerful tool to tackle these questions [17–20]. Therefore, the monophyly of Steganinae remains dubious.

In this study, we inferred a phylogenomic hypothesis for Drosophilidae based on 1,028 orthologous genes automatically annotated with BUSCO [21]. Our molecular alignment is approximately 140 times larger than the longest one previously used to infer deep drosophilid relationships [15], and we included Drosophilinae and Steganinae genera that formed paraphyletic clades in previous works to test the monophyly of the subfamilies (e.g., *Scaptodrosophila, Chymomyza* and *Phortica* [15]). We found that, despite strong gene tree heterogeneity, all used species tree methods consistently support the monophyly hypothesis when using the 1,028 genes dataset. In contrast, we recovered Steganinae as a paraphyletic group when using the concatenation approach to infer a species tree from a smaller set of ten manually annotated genes that are commonly used in phylogenetics. This result stresses how previous Drosophilidae phylogenetic studies based on smaller datasets may have been biased by gene tree heterogeneity.

## Results

### Gene tree heterogeneity

We used genomic sequences from ten species. Besides using the reference genomes of *Drosophila melanogaster* and *Drosophila virilis*, we sequenced and assembled the genomes of four species and assembled the reads downloaded from NCBI-SRA of the remaining four (see “Methods” for details).

The eight Drosophilidae species belong to seven genera: five from the subfamily Drosophilinae (*Drosophila melanogaster*, *Drosophila virilis, Scaptodrosophila lebanonensis, Colocasiomyia xenalocasiae* and *Chymomyza amoena*) and three from the subfamily Steganinae (*Phortica variegata, Rhinoleucophenga cf. bivisualis* and *Cacoxenus indagator)*. As an outgroup, we used two Ephydridae species that had been previously sequenced, *Ephydra hians* and *Ephydra gracilis*. The Drosophilidae and Ephydridae families are closely related; both belong to the Ephydroidea superfamily. Relationships among Ephydroidea families are uncertain, although the monophyly of the superfamily is well supported [22, 23].

**Fig. 1:**
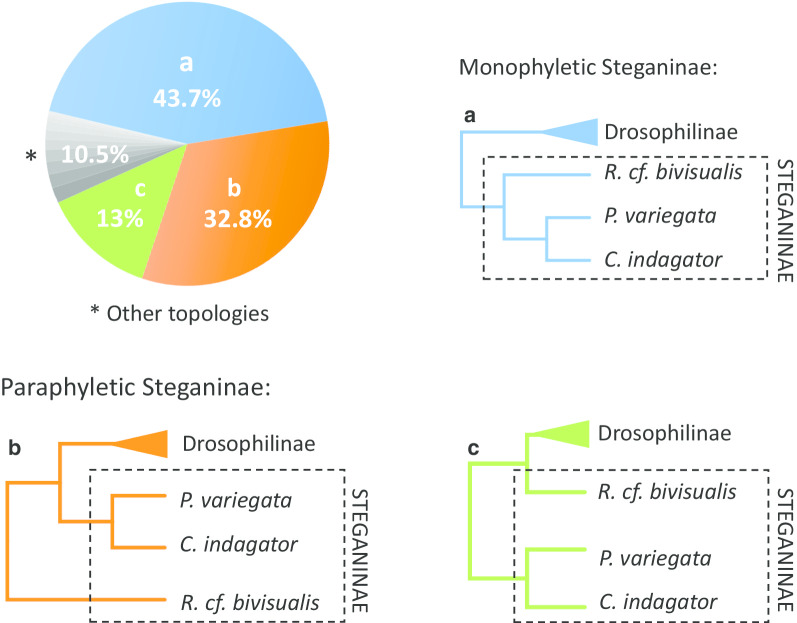
Distribution of gene trees supporting Steganinae monophyly or paraphyly. Pie chart colors relate to the color of the gene tree topologies. Of the 1,028 gene trees analyzed, 46.7% recovered the Steganinae subfamily as monophyletic and 53.4% recovered it as paraphyletic. Among the monophyletic-Steganinae trees, the predominant topology (**a**, in blue) grouped *Rhinoleucophenga cf. bivisualis* as the sister group of *Phortica variegata* + *Cacoxenus indagator* with a frequency of 43.7% of the total gene trees*.* Among the paraphyletic-Steganinae trees, two topologies were recovered with higher frequency, accounting, respectively, for 32.8% (**b**) and 13% (**c**) of the total gene trees. All the other possible topologies occurred at low frequencies, accounting together for 10.5% of the total gene trees. For further information regarding the recovered topologies, see Fig. S1 (Additional file 1)

We inferred 1,028 maximum-likelihood trees from genes annotated from genomic sequences using BUSCO [21] (see “Methods” for details). Steganinae was recovered as a monophyletic group in 46.7% of the gene trees. Among these trees, the topology that grouped *Cacoxenus indagator* and *Phortica variegata*, placing *Rhinoleucophenga cf. bivisualis* as the first lineage to diverge within Steganinae, was the most frequent (43.7% of the total) (Fig. 1, topology A). The two monophyletic-Steganinae alternative arrangements (*Phortica variegata* + *Rhinoleucophenga cf. bivisualis* and *Cacoxenus indagator* + *Rhinoleucophenga cf. bivisualis*) were much less frequent and occurred in similar proportions (1.6% and 1.4% of the total of gene trees, respectively) (Additional file 1, Fig. S1, topologies E and F). This pattern is compatible with statistical noise and/or incomplete lineage sorting (ILS) in ancient divergences within the Steganinae subfamily.

However, 53.4% of the gene trees from the phylogenomic dataset recovered Steganinae as paraphyletic. There were 10 different types of paraphyletic-Steganinae trees that recovered Drosophilinae monophyly (Additional file 1, Fig. S1, topologies B–D and G–M), with two of them being far more common than the others (Fig. 1, topologies B and C). The arrangement placing *Rhinoleucophenga cf. bivisualis* as the first lineage to diverge, followed by a lineage containing both the clade *Phortica variegata* + *Cacoxenus indagator* and Drosophilinae*,* was the most frequent, representing 32.8% of the total gene trees (Fig. 1, topology B). The second most frequent topology recovering Steganinae as paraphyletic, representing 13% of the total trees, places *Phortica variegata* + *Cacoxenus indagator* as the first lineage to diverge within Drosophilidae, followed by the split of *Rhinoleucophenga cf. bivisualis* and the Drosophilinae clade (Fig. 1, topology C). All the remaining paraphyletic-Steganinae topologies that recovered Drosophilinae monophyly occurred at low frequencies, accounting together for 6.1% of the total number of recovered trees (Additional file 1, Fig. S1, topologies D and G–M). Finally, only 1.5% of the total gene trees recovered both Drosophilinae and Steganinae as paraphyletic.

To further investigate the main causes of gene tree heterogeneity, we reduced the analysis to a four-species problem (e.g., [24, 25]) by removing species with undoubtful or nonpivotal phylogenetic positions (see “Methods”—“Phylogenetic analyses” section). Namely, we used *P. variegata* (representing the clade *Phortica* + *Cacoxenus*), *D. melanogaster* (representing the Drosophilinae subfamily), *Rhinoleucophenga cf. bivisualis*, and *E. hians* (representing the outgroup Ephydridae).

**Fig. 2:**
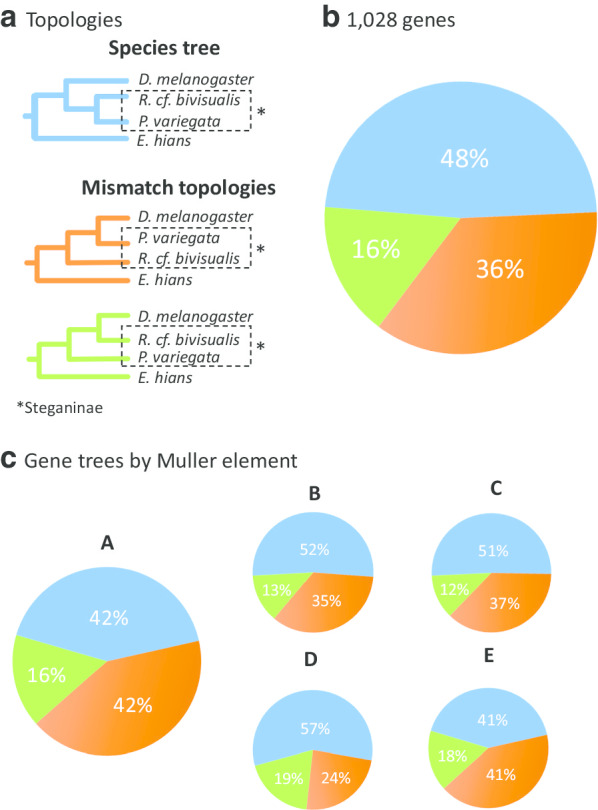
The frequency of gene tree topologies for a four-species problem. Pie chart colors relate to the color of the gene tree topologies. The most frequent gene tree (in blue) matched the species tree inferred based on the full dataset (10 species) and recovered the Steganinae subfamily as monophyletic. Mismatch topologies are shown in orange and green. **a** The species tree and the mismatch topologies; **b** frequency of the gene tree topologies estimated from the phylogenomic dataset. **c** Distribution of the gene trees by Muller element (correspondence to *D. melanogaster* chromosomes: A-X, B-2L, C-2R, D-3L, D-3R, E-4)

Under this four-species approach, the most frequent gene tree topology recovered Steganinae as monophyletic (47.7%; Fig. 2, blue tree). The other two less frequent topologies recovered Steganinae paraphyly with distinct frequencies. The grouping of *Drosophila melanogaster* and *Phortica variegata* was far more common (36.3% of gene trees; Fig. 2, orange tree) than the clustering of *Drosophila melanogaster* and *Rhinoleucophenga cf. bivisualis* (16% of gene trees; Fig. 2, green tree). Note that similar proportions of the "mismatch topologies" is a hallmark of ILS (e.g. [26, 27]). Thus, this imbalance suggests the occurrence of other phenomena, such as some level of asymmetric introgression among ancestral drosophilid branches. A possible approach used to test this hypothesis is to analyze the chromosomal location of genes (we used the *D. melanogaster* location because synteny groups known as Muller elements are very well conserved in *Drosophila* [28]). The rationale is that gene flow between closely related species tend to be much reduced in the X chromosome (Muller element A) [18, 29], leading to the expectation that a more balanced number of topologies mismatching the species tree would be found in the X-linked genes. However, an analysis of the mismatched topologies by chromosome failed to disclose any particularity of the X-linked genes (Fig. 2c).

### Species tree inference

**Fig. 3:**
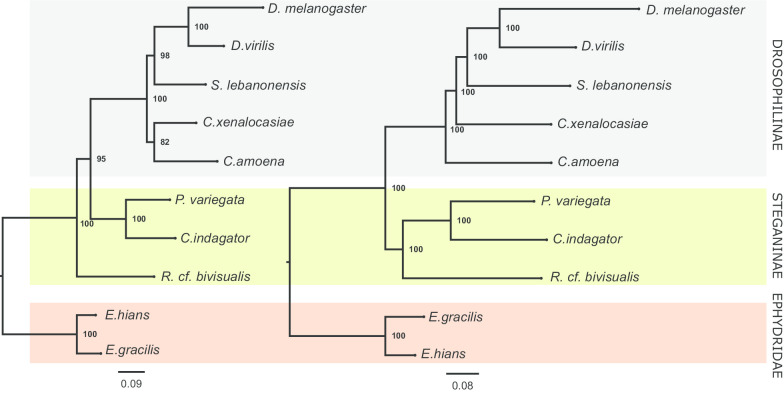
Species tree inferred with the concatenation approach in IQTree with the 10-G (left) and the phylogenomic (right) datasets. Only the phylogenomic dataset recovered the Steganinae subfamily as monophyletic. Bootstrap values are shown on nodes

**Fig. 4:**
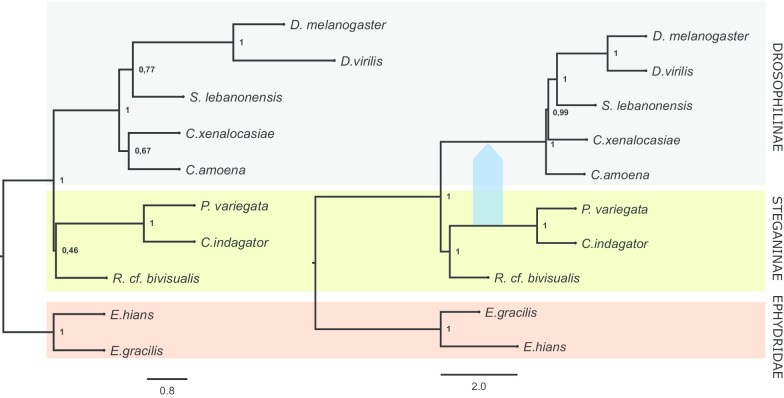
Species tree inferred in ASTRAL with the 10-G (left) and the phylogenomic (right) datasets. Both datasets recover the Steganinae subfamily as a monophyletic. Local posterior probabilities are shown on nodes. The blue arrow represents a reticulation event between the ancestral lineages of the Drosophilinae and *Phortica* + *Cacoxenus* with inheritance probability of 4.5% (inferred from the phylogenomic dataset with PhyloNet)

The concatenation approach recovered Steganinae as monophyletic in the phylogenomic dataset but not in a manually annotated set of 10 genes (10-G) commonly used in phylogenetic studies (Fig. 3—see “Methods” for further information about the 10-G dataset). However, the multispecies coalescent (MSC) approach of ASTRAL [30] recovered Drosophilinae and Steganinae monophyly for both the phylogenomic and 10-G datasets (Fig. 4). Therefore, employing the MSC model consistently led to the same inferred species tree regardless of the size of the dataset (phylogenomic or 10-G), although the 10-G analysis showed lower support for deeper nodes, such as for the Steganinae crown group (local posterior probability was 0.46 in the 10-G dataset and 1.0 in the phylogenomic dataset). Of note, the bootstrap value for the paraphyletic Steganinae was high in the phylogeny based on the 10-G concatenated dataset (0.95).

Due to the high heterogeneity and unbalance in gene trees frequency, we were prompted to infer a species network accounting for both ILS and reticulation nodes (e.g., hybridization) in PhyloNet [31]. This analysis recovered the same phylogenetic relationships as the ASTRAL and phylogenomic concatenation approaches: the monophyly of Steganinae and Drosophilinae. Additionally, it suggested that introgression events occurred between the ancestral lineages of Drosophilinae and *Phortica* + *Cacoxenus* (Fig. 4, blue arrow).

## Discussion

Drosophilinae and Steganinae subfamilies are considered monophyletic in most *Drosophila* reference books based on morphological data (e.g., [4, 8–10]), but recent molecular phylogenies have questioned it (e.g. [13–15])*.* Two main findings of this study may help to settle this. First, by using different methods for species tree inference and analyzing a Drosophilidae phylogenomic dataset that included species previously recovered as forming paraphyletic groups, we consistently found both Steganinae and Drosophilidae to be monophyletic. Second, there was a large amount of gene tree heterogeneity in the early divergences within the Drosophilidae family, which likely explains the conflicting results from previous studies, which were based on a small number of genes.

Of note, our phylogenomic dataset was ~ 140 times larger than the largest data studied previously [15] in terms of genomic sampling, and we only included genes that are present in all species, while previous studies used alignment matrices with extensive missing data (*e.g.*, [13, 14]), which may have introduced bias [32, 33]. Furthermore, we used genera that were previously recovered as members of groups that violated the monophyly of both subfamilies (e.g.,* Scaptodrosophila, Chymomyza* and *Phortica* [15]) and added two new Steganinae genomes (*Cacoxenus indagator* and *Rhinoleucophenga cf. bivisualis*) that were not previously studied.

### Species tree inference: Drosophilinae and Steganinae monophyly

For the phylogenomic dataset, all phylogenetic inference methods recovered the same species tree topology (Figs. 3, 4), mostly with high values of node support (bootstraps and posterior probabilities of 100%). Therefore, despite considerable gene tree heterogeneity, our data consistently led to a species tree that supports the monophyletic status of both Steganinae and Drosophilinae subfamilies presented in most Drosophilidae studies [4, 8–10, 16]. Moreover, it recovered *Cacoxenus* and *Phortica* as sister groups as well as a clade containing both as a sister lineage of *Rhinoleucophenga*. This result is consistent with previous morphology-based Steganinae phylogenies and the current taxonomic classification of Steganinae in which *Cacoxenus* and *Phortica* are placed within the subtribe Gitonina of the Gitonini tribe and *Rhinoleucophenga* is placed within the Acletoxenina subtribe of the Gitonini tribe [7, 34]. This relationship within Steganinae is also supported by mitochondrial data (partial cytochrome c oxidase subunit 1 sequences; ~ 700 base pairs) that grouped first *Phortica* and *Cacoxenus* and then *Gitona*, which is thought to be closely related to *Rhinoleucophenga* [13]*.*

For the 10-G dataset, the MSC method recovered the same species tree topology as the one recovered with the phylogenomic data (Fig. 4). However, concatenation resulted in a paraphyletic Steganinae, placing *Rhinoleucophenga cf. bivisualis* as the first lineage to diverge, followed by a lineage containing both the clade *Phortica variegata* + *Cacoxenus indagator* and the Drosophilinae. This topology is identical to the second most frequent gene tree of the phylogenomic dataset (Fig. 1, topology B). Concatenation has been criticized as an approach to infer species trees from small datasets that show great gene tree discordance due to ILS; in these cases, the MSC framework performs better in recovering the correct species tree [35]. Our results support this view. In our 10-G analysis, the 10 genes had different sizes ranging from 930 bp (*eve*) to 3,717 bp (*ptc*); five of them supported Steganinae paraphyly with the same topology as the one inferred in the concatenation approach (Fig. 3, left tree). The other five genes recovered alternative topologies with just three supporting Steganinae monophyly. This composition biased the species tree inference, resulting in a species tree distinct to the one obtained with the MSC and phylogenomic data. The same issue probably was the core of previous disagreements on the monophyly of Steganinae (see below). Further studies that consider a broader taxonomic sampling will be needed to better elucidate the early branching pattern of drosophilids and to confirm the monophyly of both subfamilies.

### Gene tree heterogeneity, ILS, and introgression

Comparing the phylogenetic trees inferred with 1,028 different genes, we found considerable heterogeneity in topologies with three topologies accounting for 89.5% of the estimated trees. The most frequent recovered Steganinae and Drosophilinae as monophyletic clades, and the other two recovered Steganinae as paraphyletic. High gene tree heterogeneity has been observed in many taxa. In Diptera, the best studied cases were reported in the *Drosophila melanogaster* clade [27] and in malaria vectors from the *Anopheles* genus [18]. This phenomenon is believed to result mainly from two biological events: ILS, a process by which ancestral polymorphisms persist through species divergences, and gene flow across species boundaries (i.e., introgression) [36–38]. Our results suggest that both processes played a role during the ancient radiation of Drosophilidae (below).

ILS is the simplest explanation for gene tree heterogeneity in the sense that it is a consequence of evolutionary forces that operate in all populations (mutation and genetic drift). Since introgression may be reduced in the sex chromosomes [18, 29, 39], the similar results obtained in our four species analysis for genes from the autosomal (Muller elements B–E) and X (Muller element A) chromosomes provide indirect support for ILS (Fig. 2c). Furthermore, ILS effects are stronger when speciation events occur in short intervals and populations have large effective population sizes, and further indirect support came from the previous findings that there were many episodes of rapid radiation along the evolution of flies [15, 22, 40] and the suggestion that ecological speciation was the major process in early drosophilid divergences due to empty niches [6]. Hence, our results suggest ILS as a main factor that is responsible for the incongruence between gene trees and species trees.

However, if a stochastic phenomenon, such as ILS, was the only source of gene tree heterogeneity, one would expect that the two “mismatch topologies” recovering Steganinae as paraphyletic (Fig. 1, topologies B and C) would be equally frequent, as has been observed in the classical case of *Homo*-*Gorilla*-*Chimpanzee* [26]. However, this was not the case (Fig. 1), suggesting that another source of discordance may be inflating the number of gene trees that recover one of the two alternative topologies. In fact, when estimating a phylogenetic network that accounts for introgression, we inferred a reticulation node, suggesting gene flow between ancestral lineages of Drosophilinae and *Phortica* + *Cacoxenus*. Interspecific gene flow has been already reported in many insect clades and likely played an important role in the adaptive radiation of recently diverged lineages (e.g., [18, 41–43]) and even of distantly related ones [44]. In dipterans, post-speciation gene flow has also been reported in the *Anopheles gambiae* species complex [18] and among species within the *Drosophila* genus, such as the cactophilics *Drosophila mojavensis* and *Drosophila arizonae* [43] and the sister species *Drosophila pseudobscura* and *Drosophila persimilis* [45]*.*

In the *Anopheles gambiae* species complex, interspecific gene flow led to an unresolved relationship among the species *Anopheles arabiensis, Anopheles coluzzi* and *An. gambiae* for many years [46, 47]. Recently, compelling genomic evidence has shown that a robust species tree can be reconstructed from a small section of the X chromosome, which is not prone to introgression (probably because it carries the genes that maintain reproductive isolation) [18]. Lack of introgression in the X chromosome when compared to autosomes has also been observed among *Drosophila* species [48], and these results led us to investigate if the three distinct gene tree topologies obtained in our four-species analysis were distributed differently among chromosomes. We found no evidence of such pattern (Fig. 2c), suggesting that either introgression was not a major factor in early Drosophilidae evolution and the distinct frequency of topologies may have another explanation or it occurred without leaving a distinctive pattern among X and autosomal chromosomes. A third explanation is that, since the supposed introgression occurred in the early diversification of Drosophilidae, which was dated around the K-Pg boundary [15], the signal regarding these events is no longer observable when looking into the distribution of gene topologies among chromosomes. It will be interesting to revisit this problem using more taxa and more contiguous genome assemblies, which would allow the use of synteny information (e.g. [27]).

### BUSCO as a tool for phylogenomic studies

The most standard approach for phylogenomic studies in previously unsequenced species involves genome sequencing, genome annotation and ortholog identification. Genome sequencing, of course, cannot be circumvented and, as our results exemplify, is inexpensive and technically easy if one wants only gene sequences (without fully assembled chromosomes), as Illumina sequencing offers an excellent cost–benefit solution. The last two steps, in particular genome annotation, are technically challenging [49, 50] and may be outside the reach of many labs interested in phylogenetics. By solving these two steps in a simple way for the end-user, BUSCO has become a very worthwhile tool for phylogenomic studies [51], as suggested by our results. The *Drosophila* genome contains ~ 13,000 genes, but BUSCO attempts to annotate only a subset of it, which corresponds to the 27,99 single copy ortholog genes conserved among Diptera. This is reasonable for phylogenomic purposes because hundreds of genes usually provide enough information to estimate species trees. Furthermore, the subset used by BUSCO is enriched in genes that are the most valuable for phylogenetic inference: single copy orthologs, which are present in most of the target group species (Diptera in our case), and at least moderately well conserved sequences. However, we found that it is important to check and correct BUSCO results (i.e., the gene annotations) for the presence of artifacts, such as paralogs and CDS starting at frames + 2 or + 3. We show that this can be easily achieved by using standard phylogenetic tools and simple scripts. Thus, we think that the approaches described here to improve BUSCO-derived datasets will be useful to other researchers planning to use BUSCO in phylogenomic studies.

## Conclusions

Our study corroborates the monophyletic-Steganinae hypothesis and helps clarify the possible causes of previous disagreement on the matter: the considerable heterogeneity of gene tree topologies. Such heterogeneity may have been caused primarily by ILS but also by introgression, as recovered by the phylonetwork analysis. To further investigate the monophyletic status of the subfamilies and the causes and consequences of gene tree heterogeneity in Drosophilidae, it will be interesting to invest future efforts in expanding taxon sampling by including Steganinae genera that represent its diversity of tribes and subtribes and other genera that are currently considered to have diverged early in Drosophilinae diversification. Furthermore, future studies would also benefit from the improvement of contiguity of genomic assemblies provided by long read technologies, which would permit further testing of the introgression hypothesis by analyses of gene synteny. Finally, we believe that our protocol for identifying, correcting and removing potential BUSCO misannotations will be useful in improving the application of this software as a tool for phylogenomic studies.

## Methods

### Species sampling and genome sequencing and assembling

The sources of the sequences for each species are summarized in Table S1 (Additional file 2) and Additional file 3. *D. melanogaster* and *D. virilis* were previously sequenced with Sanger technology and assembled with the appropriate assemblers [52, 53]. *Phortica variegata, Scaptodrosophila lebanonensis, Ephydra hians* and *Ephydra gracilis* were sequenced using Illumina technology by Vicoso and Bachtrog [54], and the raw reads were downloaded from NCBI-SRA. We sequenced the remaining four species (*Cacoxenus indagator*, *Chymomyza amoena, Colocasiomyia xenalocasiae* and *Rhinoleucophenga cf. bivisualis*) as follows. DNA was extracted from one individual or a pool of male flies preserved in − 20 °C ethanol using the Puregene DNA kit (Qiagen) following the manufacturer’s recommendations. Illumina paired-end DNA-seq libraries with a fragment size of 350 bp were produced and sequenced at Macrogen (Korea) with HiSeq 2000. We deposited the sequences in GenBank under Accession Numbers SRR12717851, SRR12717854, SRR12717853 and SRR12717852, respectively. All Illumina datasets were assembled with SPADES v. 3.9.0 or v. 3.11.1 [55]. See Table S2 (Additional file 2) for further information about the genome assemblies.

After assembling the genomes, we proceeded to automatic and manual ortholog annotation to obtain two datasets for phylogenetic analyses. These procedures are detailed in the next sections.

### Automatic gene annotation and identification of orthologs

We ran BUSCO v3 [21] using the default parameters with the Diptera reference set of orthologs (odb9, downloaded from https://busco.ezlab.org/ on March 22, 2018; 2,799 genes) to assess the quality of the assembled genomes (Table S3, Additional file 2) and to obtain a dataset of genes that are present in the 10 species used in this study. BUSCO (Dipteran database) searches for a set of 2,799 genes that are well conserved and present in nearly all Diptera genomes. Although the absence of some genes from this set in a given genome may be due to true gene loss, more frequently it indicates assembly problems; thus, BUSCO can be used to infer assembly quality. In all Drosophilidae species used here, BUSCO successfully identified more than 90% of the Dipteran database proteins. In the outgroup species of the Ephydridae family, the proportion of fragmented and missing proteins was higher, and the amount of identified proteins was lower: 85.2% for *Ephydra hians* and 83.7% for *Ephydra gracilis.* Genome coverage for these two species were low (*E. hians*: 7.3 ×; *E. gracilis*: 4.1 ×), but it was even lower for *Phortica variegata* (2.7 ×), where the assembly had a higher BUSCO score (93.3%). To put these BUSCO scores in context, they frequently are > 95% for model organisms, whereas for nonmodel organisms, this value was reportedly lower (50–95%), depending on factors such as genome size, the amount of repetitive elements and the taxonomic position of a species [56]*.* Thus, all genomes had at least a reasonable quality, and eventual missing genes would not bias the analysis because we only used genes identified in all 10 genomes (see below).

In addition to genome assemblies, phylogenomic analyses require ortholog identification. Although BUSCO was originally designed to estimate the completeness of the genome assemblies, it is also suitable for phylogenomic purposes [51] because it simultaneously identifies the orthologs of a conserved set of genes and extracts their coding sequences from the genome sequences. However, we noticed that the automatic annotation of the orthologs made by BUSCO had errors that could bias the phylogenetic inference. Such problems were detailed and corrected as described below.

### Correction of BUSCO artifacts

From the initial set of 2,799 genes, 1,603 were retrieved by BUSCO in all 10 genomes. In some genes, the ortholog present in one species was much smaller or larger than the others, indicating annotation or orthology problems. Therefore, we removed the genes with the coefficient of variation of protein size larger than 10%, resulting in our initial dataset of 1,100 genes. However, during the initial phylogenetic analysis, we detected problems in this dataset. In some cases, we could correct the error, and when this was not possible, we removed the affected gene (in all 10 species) from the analyses (Additional file 4). These procedures are detailed below.

We initially noticed that some gene trees have one abnormally long terminal branch, suggesting an annotation problem (e.g., Fig. S2, Additional file 5). We searched for these trees by examining the distribution of the total sum of branch lengths (SBL) as a proxy of discrepancy (Fig. S3, Additional file 6). Outlier gene trees (e.g., SBL > 30) were manually inspected, and we found that many of them were caused by an undesirable feature in BUSCO annotation: some coding sequences were extracted with frame + 2 or + 3, instead of + 1, and hence could not be properly aligned with the Perl script translatorx_vLocal.pl [57], which translates them to the protein assuming a frame of + 1. An in-house awk script (fix_busco_CDS_frame.txt, Additional file 7) was used to correct this problem, resulting in a set of 1,110 genes that had a more homogeneous distribution of SBL (Fig. S4, Additional file 8). However, some gene trees still seemed to be outliers, and, by examining more carefully the trees with SBL > 10, we found that all of them had one or two species in which a paralog was annotated instead of the true ortholog. In all cases, this error was caused by assembly fragmentation: BUSCO missed the true ortholog because it was broken into two or more scaffolds (Fig. S5, Additional file 9).

The two artifacts mentioned above were quite easy to spot, but we could not exclude the possibility that less obvious problems might have biased the result. To address this possibility, we removed from the dataset the genes with the top 5% highest SBL or the top 5% highest root-to-tip variance (Fig. S6, Additional file 10). These two measures were partially correlated (Fig. S6, Additional file 10), and we hoped that combining them would improve the removal of potentially erroneous gene trees. This led to a final dataset of 1,028 genes. Importantly, the annotation correction or gene removal was based solely on the heterogeneity of branch lengths and was completely blind to the Steganinae monophyly vs. paraphyly question.

### Manual annotation of a 10-gene dataset

As a control, besides using automatic annotation, we manually annotated a smaller dataset of 10 genes, some of which were used in previous drosophilid phylogenetic studies (e.g., [14–16]): Patched (*ptc*), Even-skipped (*eve*), Ebony (*eb*), Engrailed (*en*), Dopa-decarboylase (*ddc*), Notum (*notum*), Wingless (*wg*), Hedgehog (*hh*), Distal-less (*dll*) and Amylase Related (*Amyrel*). This approach aimed to address the potential bias introduced by the automatic ortholog identification and gene annotation and to emulate the gene sampling from the previously mentioned phylogenetic studies, which were based on small datasets of hand-curated genes. These 10 genes were annotated as follows. The *D. melanogaster* protein sequences were obtained from FlyBase [58] and used in a local TblastN search [59] against our databases of drosophilids assembled genomes to identify the scaffold that contained the gene. This scaffold (or the subregion that contains the gene) was computationally annotated by GeneWise [60] and NAP [61], and the output files were manually curated to obtain the final annotation (i.e., identify the intron–exon structure, initial methionine, stop codons, and the coding sequence). This resulted in a set of 100 curated coding sequences (CDS) with 10 for each of the 10 species (a total of 198,030 bp). This dataset was referred to in this study as 10-G.

### Phylogenetic analyses

The procedures used for gene tree and species tree inference are summarized in Fig. S7 (Additional file 11). Resources used to align an concatenate sequences are provided in Additional files 4, 12 and 13.

The Perl script translatorx_vLocal.pl [57] was used to align nucleotide sequences based on the protein sequences they encode with the options -p F -g 1 (to select MAFFT v7.394 as the aligner software and use GBlocks v0.9 to remove poorly aligned regions, respectively). Then, maximum-likelihood gene trees were inferred with IQ-TREE 1.6.1 [62] using the best-fit substitution model for each gene [63].

Afterwards, three approaches were used to infer the Drosophilidae species tree: concatenation, the traditional multispecies coalescent (MSC) framework that accounts only for ILS [30], and an MSC framework that accounts for both ILS and introgression ("reticulation" [31]).

In the concatenation approach, phylogenetic inference was conducted with IQ-TREE 1.6.1 [62] for both the phylogenomic and the 10-G datasets. For the phylogenomic dataset, we applied a data partitioning scheme by gene with the best-fit substitution models selected for each gene independently; for the 10-G dataset, we used the best partition-scheme and corresponding best-fit models selected by PartitionFinder in IQ-TREE [63, 64]. For both datasets, 1,000 bootstrap replicates were obtained in all analysis. Importantly, phylogenetic inference was also conducted with the GHOST model [65] in IQ-TREE to evaluate whether ignoring heterotachy would bias the results. We report the results based only on nonheterotachy models because they were the same as when we employed the GHOST mixture model.

In the MSC approach, species trees for both datasets (phylogenomic and 10-G) were inferred using ASTRAL 5.6.1 [30]. A phylogenetic network that accounted for introgression was inferred with PhyloNet [31] for the phylogenomic dataset. We used the “InferNetwork MPL” command and set the maximum number of reticulations allowed as one.

As described in the “Results” section, we found high levels of gene tree heterogeneity regarding Steganinae monophyly/paraphyly. To further investigate the possible sources of this heterogeneity, we made additional analyses by reducing our phylogenomic dataset to a four-species problem as follows. The monophyly of Drosophilidae, Drosophilinae and the clade *Cacoxenus indagator* plus *Phortica variegata* was recovered in the majority of our gene trees. These lineages were also widely regarded as well established by several studies [4, 7, 14–16, 66]. Thus, we inferred gene trees considering a reduced set of four species: *Rhinoleucophenga cf. bivisualis*, *Phortica variegata* (representing the clade *Cacoxenus indagator* plus *Phortica variegata*)*, Drosophila melanogaster* (representing the Drosophilinae subfamily) and *Ephydra hians* (outgroup). There were only three possible topologies when considering four-species rooted trees. Thus, we were able to evaluate the frequencies of the two mismatch gene trees, which should have been equivalent under the absence of reticulate evolution (i.e., introgression). To investigate the possibility of a biased support of gene topologies between different chromosomes, as already reported in other biological groups [18, 67], we split the dataset according to the gene location on the autosomal (Muller elements B–E) and X (Muller element A) chromosomes in *Drosophila melanogaster*. Since chromosomal arm nomenclatures can vary among species [68, 69], we adopted the Muller standard nomenclature [70] to identify linkage groups.

## Supplementary information

**Additional file 1: Fig. S1:** Topologies obtained from the phylogenomic dataset and their frequencies. Topologies A, E and F recovered the Steganinae subfamily as monophyletic and account together for 46.7% of the total gene trees. The remaining trees recovered the Steganinae as paraphyletic with distinct topologies. Only 1.5% of the trees recovered the Drosophilinae subfamily as a paraphyletic.

**Additional file 2: Table S1.** Sources of the genome assemblies used for phylogenetic analysis. **Table S2:** Drosophilidae genome assemblies’ statistics. **Table S3.** BUSCO results for the 10 species. The total number of single-copy ortholog genes present in the Diptera database was 2,799.

**Additional file 3:** Source of sequenced samples.

**Additional file 4: BUSCO_cleaning_pipeline.txt**: shell pipeline for curating BUSCO results.

**Additional file 5: Fig. S2.** Example of a gene tree with abnormally long terminal branches (sum of branch lengths: 101.1), suggesting a BUSCO annotation problem. In this case, the gene CG7739 (in *D. melanogaster*) was annotated out of frame for *Ephydra hians* and *Phortica variegata*, resulting in a problematic alignment and, consequentially, an inaccurate gene tree.

**Additional file 6: Fig. S3.** Histogram showing the distribution of the total sum of branch lengths (SBL) for each of the 1,100 genes trees. Phylogenetic trees were inferred under the Maximum Likelihood method for each of the genes annotated by BUSCO, and their total sum of branch lengths (SBL) was used to identify discrepant trees. We found a bimodal distribution, and found that abnormally high SBL, such as those in the right peak, were generated by annotation errors.

**Additional file 7: fix_busco_CDS_frame.txt**: BUSCO produced some CDS with frame + 2 or + 3, which created problems in the following analysis with translatorX. This awk script corrects the reading frame of all CDS to + 1.

**Additional file 8: Fig. S4.** Histogram showing the distribution of the total sum of branch lengths (SBL) of each of the 1,100 genes trees after fixing sequences with frame shifts. This approach reduced the number of sequences with abnormally high SBL, but there were still gene trees with discrepant values.

**Additional file 9: Fig. S5.** Example of a gene tree with abnormally long branch lengths (sum of branch lengths: 36.8) due to the misannotation of *Phortica variegata* ortholog related to the *D. melanogaster*’s gene CG7432 by BUSCO **(a)**. As showed in **b**, in this case, BUSCO annotated a paralogous sequence (arrow), since the orthologous one was scattered in two scaffolds (in red).

**Additional file 10: Fig. S6.** Distribution of the variance and total sum of branch lengths of gene trees. The dashed lines indicate the 5% cutoff values established to exclude potentially problematic trees for both parameters.

**Additional file 11: Fig. S7.** Summary of the phylogenetic methods used for gene and species trees inference.

**Additional file 12: busco2multifasta.txt**: awk script that produces a multifasta file ready to be aligned based on a BUSCO list of single-copy orthologs of two or more species.

**Additional file 13: supermatrix2.txt**: awk script that concatenates alignments in MEGA format, preparing the data for a supermatrix analysis.

## Data Availability

The datasets generated and/or analyzed during the current study are available in the GitHub repository, https://github.com/GuilhermeRDias/DrosophilidaePhylogenomics.

## Abbreviations

ILS: Incomplete lineage sorting
10-G: 10-Gene dataset
NCBI-SRA: National Center for Biotechnology Information Sequence Read Archive
K-Pg: Cretaceous-Paleogene
MSC: Multispecies coalescent
SBL: Total sum of branch lengths
CDS: Coding sequence
BUSCO: Benchmarking Universal Single-Copy Orthologs

## Authors' information

This work is part of the Ph.D. thesis of GRD, from the Genetics Graduation Program at the Biology Institute of Federal University of Rio de Janeiro.
