# Supplementary figures and images for: A phylogenomic study of Steganinae fruit flies (Diptera: Drosophilidae): strong gene tree heterogeneity and evidence for monophyly
Source: BMC Evol Biol. 2020 Nov 2;20:141. doi: 10.1186/s12862-020-01703-7 (PMC7607883; doi:10.1186/s12862-020-01703-7)

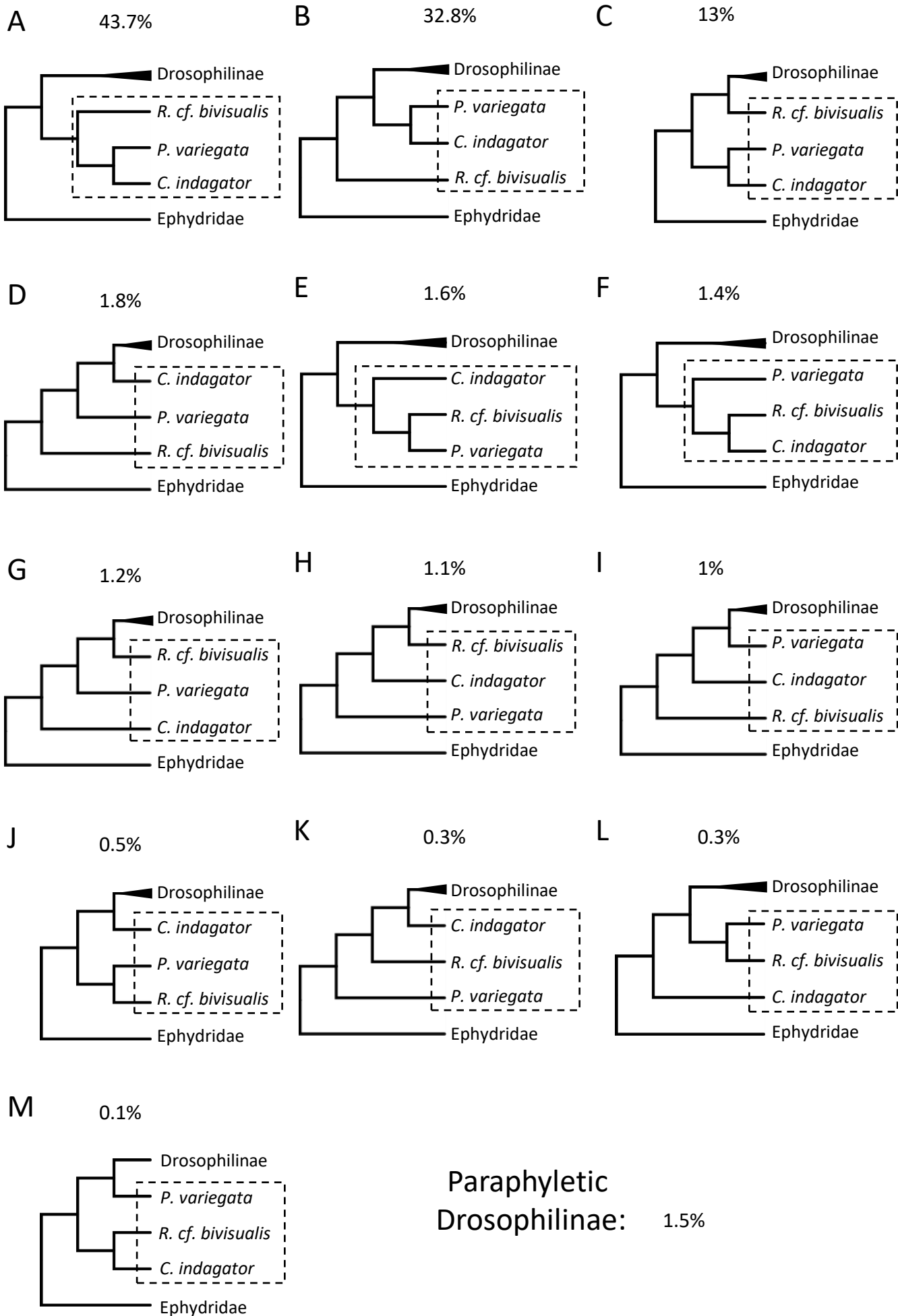

Supplement: Supplementary file 1 — Additional file 1: Fig. S1: Topologies obtained from the phylogenomic dataset and their frequencies. Topologies A, E and F recovered the Steganinae subfamily as monophyletic and account together for 46.7% of the total gene trees. The remaining trees recovered the Steganinae as paraphyletic with distinct topologies. Only 1.5% of the trees recovered the Drosophilinae subfamily as a paraphyletic. [file 12862_2020_1703_MOESM1_ESM.pdf]

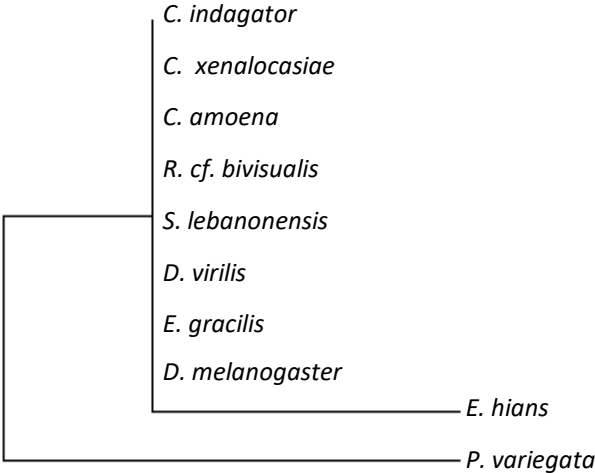

Supplement: Supplementary file 2 — Additional file 2: Table S1. Sources of the genome assemblies used for phylogenetic analysis. Table S2: Drosophilidae genome assemblies’ statistics. Table S3. BUSCO results for the 10 species. The total number of single-copy ortholog genes present in the Diptera database was 2,799. [file 12862_2020_1703_MOESM2_ESM.pdf]

Histogram of sum

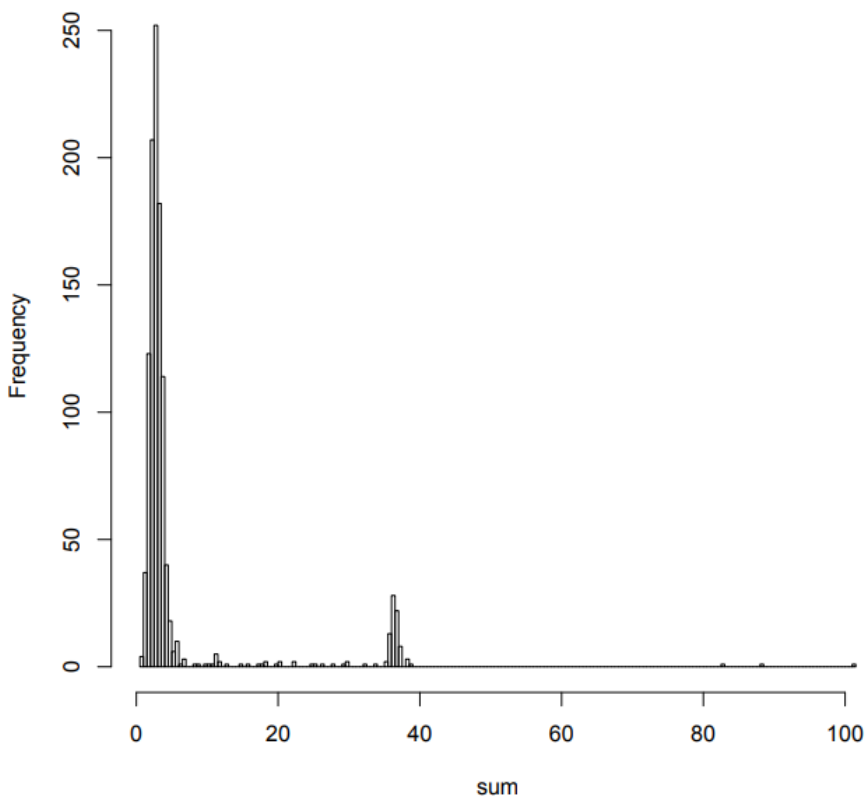

Supplement: Supplementary file 5 — Additional file 5: Fig. S2. Example of a gene tree with abnormally long terminal branches (sum of branch lengths: 101.1), suggesting a BUSCO annotation problem. In this case, the gene CG7739 (in D. melanogaster) was annotated out of frame for Ephydra hians and Phortica variegata, resulting in a problematic alignment and, consequentially, an inaccurate gene tree. [file 12862_2020_1703_MOESM5_ESM.pdf]

Histogram of sum

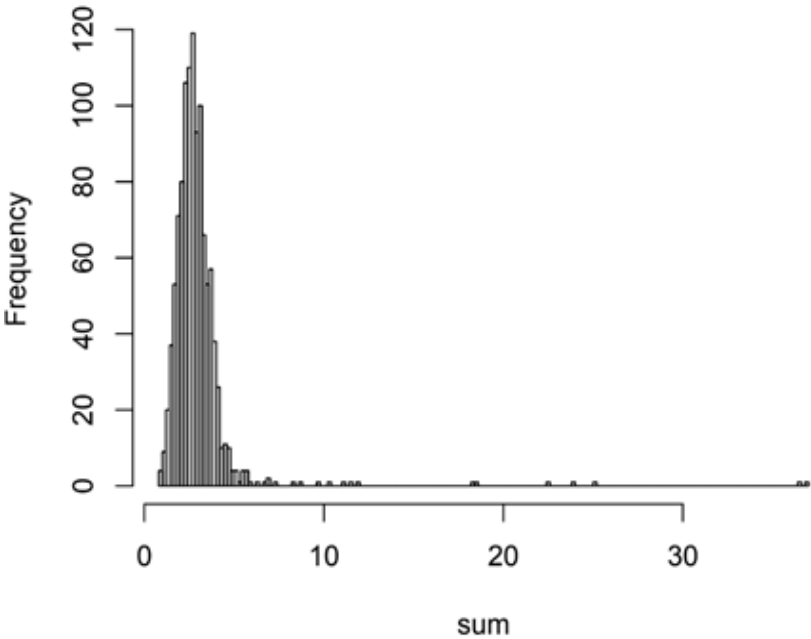

Supplement: Supplementary file 6 — Additional file 6: Fig. S3. Histogram showing the distribution of the total sum of branch lengths (SBL) for each of the 1,100 genes trees. Phylogenetic trees were inferred under the Maximum Likelihood method for each of the genes annotated by BUSCO, and their total sum of branch lengths (SBL) was used to identify discrepant trees. We found a bimodal distribution, and found that abnormally high SBL, such as those in the right peak, were generated by annotation errors. [file 12862_2020_1703_MOESM6_ESM.pdf]

A

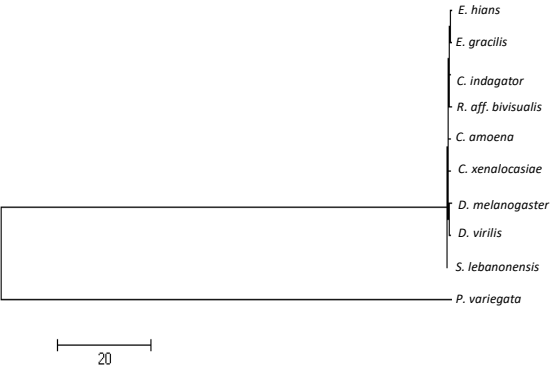

B

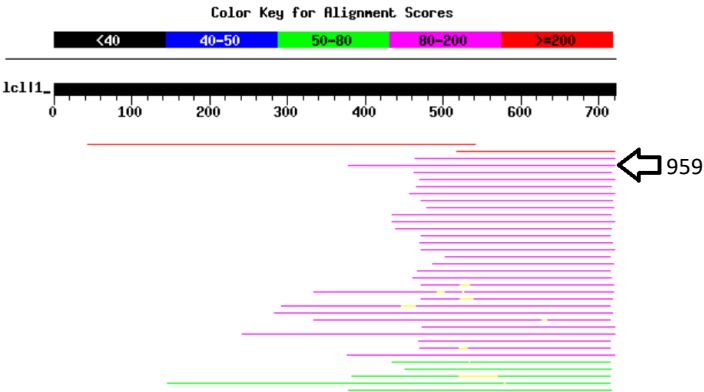

Supplement: Supplementary file 7 — Additional file 7: fix_busco_CDS_frame.txt: BUSCO produced some CDS with frame + 2 or + 3, which created problems in the following analysis with translatorX. This awk script corrects the reading frame of all CDS to + 1. [file 12862_2020_1703_MOESM7_ESM.pdf]

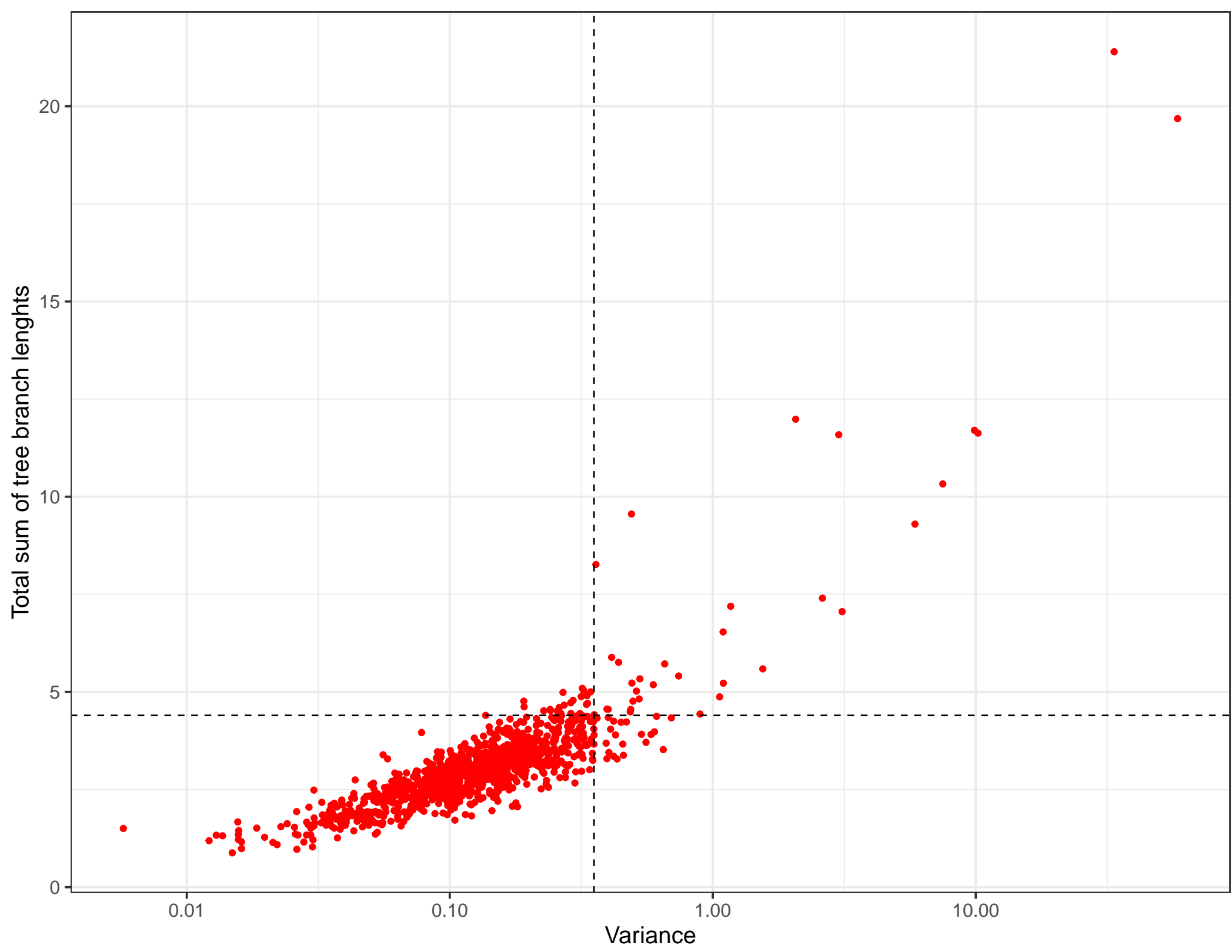

Supplement: Supplementary file 8 — Additional file 8: Fig. S4. Histogram showing the distribution of the total sum of branch lengths (SBL) of each of the 1,100 genes trees after fixing sequences with frame shifts. This approach reduced the number of sequences with abnormally high SBL, but there were still gene trees with discrepant values. [file 12862_2020_1703_MOESM8_ESM.pdf]

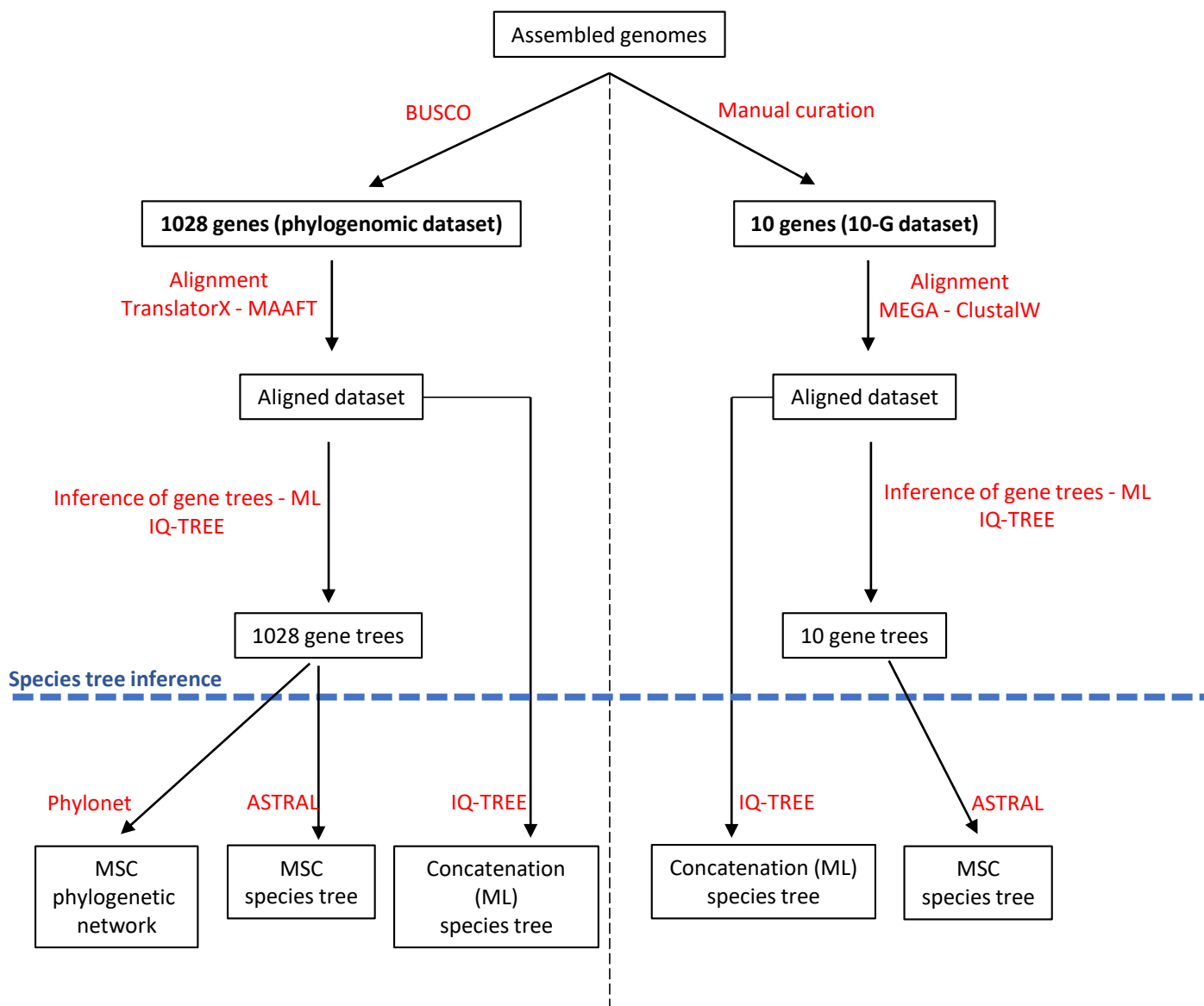

Supplement: Supplementary file 9 — Additional file 9: Fig. S5. Example of a gene tree with abnormally long branch lengths (sum of branch lengths: 36.8) due to the misannotation of Phortica variegata ortholog related to the D. melanogaster’s gene CG7432 by BUSCO (a). As showed in b, in this case, BUSCO annotated a paralogous sequence (arrow), since the orthologous one was scattered in two scaffolds (in red). [file 12862_2020_1703_MOESM9_ESM.pdf]
