# Supplementary material for: A phylogenomic study of Steganinae fruit flies (Diptera: Drosophilidae): strong gene tree heterogeneity and evidence for monophyly
Source: BMC Evol Biol. 2020 Nov 2;20:141. doi: 10.1186/s12862-020-01703-7 (PMC7607883; doi:10.1186/s12862-020-01703-7)
Supplement: Supplementary file 10 — Additional file 10: Fig. S6. Distribution of the variance and total sum of branch lengths of gene trees. The dashed lines indicate the 5% cutoff values established to exclude potentially problematic trees for both parameters. [file 12862_2020_1703_MOESM10_ESM.docx]

**Additional tables:**

**Table S1.** Sources of the genome assemblies used for phylogenetic analysis.

| **Species** | **Family** | **Subfamily** | **Strain (source)*** | **Sequencing** | **Assembly** |
| --- | --- | --- | --- | --- | --- |
| *Drosophila melanogaster* | Drosophilidae | Drosophilinae | ISO1 (BDGP) | [68] | [52, 68] |
| *Drosophila virilis* | Drosophilidae | Drosophilinae | (NDSSC) | [53] | [53] |
| *Scaptodrosophila lebanonensis* | Drosophilidae | Drosophilinae | [54] | [54] | this work |
| *Colocasiomyia xenalocasiae* | Drosophilidae | Drosophilinae | E16801 (EHIME)** | this work | this work |
| *Chymomyza amoena* | Drosophilidae | Drosophilinae | 20010-2010.01 (NDSSC)** | this work | this work |
| *Rhinoleucophenga cf. bivisualis* | Drosophilidae | Steganinae | Brasilia, Brazil – collected by Tidon, R.** | this work | this work |
| *Phortica variegata* | Drosophilidae | Steganinae | [54] | [54] | this work |
| *Cacoxenus indagator* | Drosophilidae | Steganinae | France – collected by P. Bee** | this work | this work |
| *Ephydra hians* | Ephydridae | Ephydrinae | [54] | [54] | this work |
| *Ephydra gracilis* | Ephydridae | Ephydrinae | [54] | [54] | this work |

* BDGP, Berkeley Drosophila Genome Project (USA); NDSSC, National Drosophila Species Stock Center (USA); EHIME, *Drosophila* Stocks of Ehime University (Japan).

** Further information in “Source of sequenced samples” section (Additional file 9).

**Table S2:** Drosophilidae genome assemblies’ statistics

|  | Number of contigs | Total length (bp) | Largest contig (bp) | N50 | Average coverage** | Number of contigs (>=500 bp) | Total length (>=500 bp) | Read length (bp) |
| --- | --- | --- | --- | --- | --- | --- | --- | --- |
| *Scaptodrosophila lebanonensis* | 306,315 | 213,742,963 | 198,961 | 18,272 | 10 | 26,791 | 186,025,877 | 202 |
| *Chymomyza amoena* | 575,678 | 502,039,591 | 857,774 | 11,592 | 24,6 | 103,609 | 413,728,656 | 150 |
| *Colocasiomyia xenalocasiae* | 141,820 | 266,623,368 | 169,055 | 10,455 | 13.3 | 55,733 | 243,308,510 | 100 |
| *Phortica variegata* | 86,005 | 159,533,142 | 166,446 | 12,758 | 2.7 | 25,409 | 153,128,097 | 160 |
| *Cacoxenus indagator* | 96,565 | 234,358,748 | 454,413 | 39,813 | 20 | 17,524 | 220,079,736 | 150 |
| *Rhinoleucophenga cf. bivisualis* | 292,564 | 322,942,938 | 241,760 | 9,642 | 17.2 | 91,003 | 275,608,224 | 100 |
| *Ephydra gracilis* | 107,295 | 395,606,700 | 153,157 | 9,494 | 4.1 | 68,541 | 386,258,945 | 180 |
| *Ephydra hians* | 196,554 | 441,075,528 | 115,934 | 8,253 | 7.3 | 95,762 | 419,683,199 | 180 |

** Calculated from the average coverage of the 10 larger contigs, as reported by SPAdes.

**Table S3**. BUSCO results for the 10 species. The total number of genes present in the Diptera database was 2,799.

|  | **Complete BUSCOs (C)** | | **Complete and single-copy BUSCOs (S)** | | **Complete and duplicated BUSCOs (D)** | | **Fragmented BUSCOs (F)** | | **Missing BUSCOs (M)** | |
| --- | --- | --- | --- | --- | --- | --- | --- | --- | --- | --- |
|  | **n** | **%** | **n** | **%** | **n** | **%** | **n** | **%** | **n** | **%** |
| *D. melanogaster* | 2762 | 98.7% | 2748 | 98.2% | 14 | 0.5% | 21 | 0.8% | 16 | 0.6% |
| *D. virilis* | 2728 | 97.5% | 2718 | 97.1% | 10 | 0.4% | 36 | 1.3% | 35 | 1.3% |
| *S lebanonensis* | 2610 | 93.2% | 2587 | 92.4% | 23 | 0.8% | 136 | 4.9% | 53 | 1.9% |
| *C. xenalocasiae* | 2576 | 92% | 2560 | 91.5% | 16 | 0.6% | 130 | 4.6% | 93 | 3.3% |
| *C. amoena* | 2659 | 95% | 2627 | 93.9% | 32 | 1.1% | 97 | 3.5% | 43 | 1.5% |
| *P. variegata* | 2611 | 93.3% | 2597 | 92.8% | 14 | 0.5% | 132 | 4.7% | 56 | 2% |
| *C. indagator* | 2652 | 94.7% | 2622 | 93.7% | 30 | 1.1% | 83 | 3% | 64 | 2.3% |
| *R. cf. bivisualis* | 2644 | 94.5% | 2616 | 93.5% | 28 | 1% | 113 | 4% | 42 | 1.5% |
| *E. hians* | 2386 | 85.2% | 2374 | 84.8% | 12 | 0.4% | 273 | 9.8% | 140 | 5% |
| *E. gracilis* | 2342 | 83.7% | 2329 | 83.2% | 13 | 0.5% | 314 | 11.2% | 143 | 5.1% |

**References (numbers refer to the main text)**

52. Hoskins RA, Carlson JW, Wan KH, Park S, Mendez I, Galle SE, et al. The Release 6 reference sequence of the Drosophila melanogaster genome. Genome Res. 2015;25:445–58. doi:10.1101/gr.185579.114.

53. Clark AG, Eisen MB, Smith DR, Bergman CM, Oliver B, Markow T a, et al. Evolution of genes and genomes on the Drosophila phylogeny. Nature. 2007;450:203–18. doi:10.1038/nature06341.

54. Vicoso B, Bachtrog D. Numerous Transitions of Sex Chromosomes in Diptera. PLoS Biol. 2015;13:1–22.

68. Adams MD, Celniker SE, Holt RA, Cheryl A. Evans, Gocayne JD, Amanatides PG, et al. The Genome Sequence of Drosophila melanogaster. Science (80- ). 2000;287:2185–95. doi:10.1126/science.287.5461.2185.
