# Supplementary material for: A phylogenomic study of Steganinae fruit flies (Diptera: Drosophilidae): strong gene tree heterogeneity and evidence for monophyly
Source: BMC Evol Biol. 2020 Nov 2;20:141. doi: 10.1186/s12862-020-01703-7 (PMC7607883; doi:10.1186/s12862-020-01703-7)
Supplement: Supplementary file 11 — Additional file 11: Fig. S7. Summary of the phylogenetic methods used for gene and species trees inference. [file 12862_2020_1703_MOESM11_ESM.docx]

**Source of sequenced samples:**

*Chymomyza amoena.* We used 30 adult males of The National *Drosophila* Species Stock Center strain number 20010-2010.01 (*Chymomyza amoena* Camo\wild type East Lansing, Michigan (1992)). This is a wild type strain, inbred eight generations through single brother-sister pairs by E. G. Dupim between 2014-2015.

*Cacoxenus indagator*. We used the thoraxes of 30 adult females collected by Paul Bee in France, in Villers St Bathelemy (Post-code 60650), in the region of Picardie.

*Colocasiomyia xenalocasiae*. We used 6 adult males of *Drosophila* Stocks of Ehime University strain number E16801.

*Rhinoleucophenga cf. bivisualis*. We used one adult male collected by R. Tidon in Brasilia, Brazil.
